# Augmenting Outpatient Alcohol Treatment as Usual With Online Alcohol Avoidance Training: Protocol for a Double-Blind Randomized Controlled Trial

**DOI:** 10.2196/resprot.9287

**Published:** 2018-03-01

**Authors:** Marleen KJ Bratti-van der Werf, Melissa C Laurens, Marloes G Postel, Marcel E Pieterse, Somaya Ben Allouch, Reinout W Wiers, Ernst T Bohlmeijer, Elske Salemink

**Affiliations:** ^1^ Technology, Health & Care Research Group Saxion University of Applied Sciences Enschede Netherlands; ^2^ Centre for eHealth and Well-Being Research Department of Psychology, Health & Technology University of Twente Enschede Netherlands; ^3^ Tactus Addiction Treatment Enschede Netherlands; ^4^ Addiction Development and Psychopathology Lab Department of Development Psychology University of Amsterdam Amsterdam Netherlands

**Keywords:** cognitive bias modification, alcohol, Alcohol Avoidance Training, Approach-Avoidance Task, treatment as usual, cognitive behavioral treatment

## Abstract

**Background:**

Recent theoretical models emphasize the role of impulsive processes in alcohol addiction, which can be retrained with computerized Cognitive Bias Modification (CBM) training. In this study, the focus is on action tendencies that are activated relatively automatically.

**Objective:**

The aim of the study is to examine the effectiveness of online CBM Alcohol Avoidance Training using an adapted Approach-Avoidance Task as a supplement to treatment as usual (TAU) in an outpatient treatment setting.

**Methods:**

The effectiveness of 8 online sessions of CBM Alcohol Avoidance Training added to TAU is tested in a double-blind, randomized controlled trial with pre- and postassessments, plus follow-up assessments after 3 and 6 months. Participants are adult patients (age 18 years or over) currently following Web-based or face-to-face TAU to reduce or stop drinking. These patients are randomly assigned to a CBM Alcohol Avoidance or a placebo training. The primary outcome measure is a reduction in alcohol consumption. We hypothesize that TAU + CBM will result in up to a 13-percentage point incremental effect in the number of patients reaching the safe drinking guidelines compared to TAU + placebo CBM. Secondary outcome measures include an improvement in health status and a decrease in depression, anxiety, stress, and possible mediation by the change in approach bias. Finally, patients’ adherence, acceptability, and credibility will be examined.

**Results:**

The trial was funded in 2014 and is currently in the active participant recruitment phase (since May 2015). Enrolment will be completed in 2019. First results are expected to be submitted for publication in 2020.

**Conclusions:**

The main purpose of this study is to increase our knowledge about the added value of online Alcohol Avoidance Training as a supplement to TAU in an outpatient treatment setting. If the added effectiveness of the training is proven, the next step could be to incorporate the intervention into current treatment.

**Trial Registration:**

Netherlands Trial Register NTR5087; http://www.trialregister.nl/trialreg/admin/rctview.asp?TC=5087 (Archived at WebCite http://www.webcitation.org/6wuS4i1tH)

## Introduction

### Background

Alcohol misuse is a key public health concern and is associated with a high burden of disease, which in turn contributes to considerable economic costs for both individuals and society [1]. Although addicted people are aware of the negative consequences, most continue this self-destructive behavior. This paradox can be explained by models that conceptualize addictive behavior as a “dual process”: an imbalance between relatively automatic processes and conscious/cognitive processes [2].

Recent research provides evidence that addictive behaviors are partly guided by relatively automatic processes that occur outside conscious control, making the individual respond impulsively to cues associated with the addictive substance, rather than displaying inhibitory control [3,4]. Multiple implicit cognitive biases have been shown to play a role in alcohol addiction, such as an attentional bias for alcohol-related stimuli [5], a memory bias for the automatic activation of alcohol-related associations [6], and a bias toward automatically activated action tendencies to approach alcohol [7].

Cognitive behavioral therapy (CBT) is an evidence-based treatment for a variety of disorders including alcohol use disorders. Various meta-analyses show a large effect size in treatment outcomes of patients with alcohol disorders compared to no treatment and a small but clinically significant effect when compared to other active treatments [8,9]. Although effective, CBT programs primarily target the reflective, voluntary system and leave the automatic, impulsive system mostly unaffected [10,11]. This suggests that treatment of alcohol use disorder could be improved by also focusing on those processes that are primarily automatic. Over the past decade, a set of computerized training programs have been developed with the aim of reducing automatic biases in information processing and thereby reducing psychopathology. Collectively, these programs are termed Cognitive Bias Modification (CBM) training [12-15]. Alcohol-related CBM programs have been shown to be effective in changing attentional bias [16-18], memory bias [19,20], and approach bias [21-23], which in turn is often associated with reductions in drinking behavior or reduced relapse into drinking behavior.

The current study focuses on retraining the automatically triggered behavioral tendency to approach alcohol, by using online Alcohol Avoidance Training. In a preliminary test with this CBM training among heavily drinking students, it was shown to be successful in modifying the automatic action tendencies and related memory associations, and students who were successfully trained to avoid alcohol drank less alcohol in a taste test directly after the training [22]. The first clinical trials with German alcohol-dependent inpatients showed that this training reversed the patients’ approach bias into an avoidance bias for alcohol with generalization of the training effects to other experimental tasks [21].

More importantly, compared to patients in the placebo condition, patients in the training condition showed significantly less relapse after a year. A second study in the same clinic replicated the main findings and showed that the effects on relapse were mediated by change in approach bias [23]. While these studies tested the added effect of CBM training on top of treatment as usual (TAU), recent studies examined the effect of the training as a stand-alone intervention and failed to observe positive training effects [24,25].

Because TAU for alcohol use disorders often comprises of outpatient treatment, it is relevant to study the added value of CBM outside the clinical setting. Effectiveness of CBM in an outpatient setting may be attenuated by a lower adherence as compared to an inpatient setting. However, offering CBM online at home seems to generate high adherence rates. Combining Internet-based CBM with Internet-based CBT was found to be an acceptable form of treatment delivery for patients with depression, showing full adherence to the seven CBM sessions by 81% of participants [26]. Similarly, Salemink and colleagues showed an adherence rate of 85% among patients with different anxiety disorders, completing all 8 online training sessions (45 min each) in a period of 11 days [27]. Delivering CBM online in an outpatient setting may even generate stronger effects than in a clinical setting. There is preliminary evidence that training in a relevant context improves the effectiveness of CBM training. Kuckertz et al [28] showed improved results for CBM training in anxiety patients when patients were in a state increased anxiety while undergoing the CBM training. In case of alcohol addiction, training in a relevant, real-life context might lead to better results.

We, therefore, are interested in whether the positive added effects found in clinical inpatient samples [21] with the CBM training on local desktop computers in the clinic is possible to reproduce when the CBM is administered online in an outpatient sample.

### Aims and Hypotheses

The aim of the current study is to investigate the effectiveness of online CBM Alcohol Avoidance Training as an adjunct to TAU in an outpatient treatment setting. Patients receive eight sessions of either the active or placebo version of the CBM Alcohol Avoidance Training during their TAU. The primary goal is to test the effects of this adjunct CBM on alcohol use immediately after finishing the intervention and three and six months later, by looking at the changes in the level of alcohol consumption over a week. The primary outcome measure is the percentage of the patients reaching the low-risk drinking level, defined as <22 standard units/week for men and <15 for women [29]. It is expected that more patients in the experimental condition will reach low-risk drinking level. Furthermore, it is hypothesized that the CBM intervention decreases or reverses the approach bias, and these changes are expected to mediate the effects on alcohol use [23]. An improvement in health status and a decrease in depression, anxiety and stress for patients in the training condition, compared to the placebo condition, are expected as secondary outcomes. To investigate who benefits most from the training, possible moderators will be examined. Automatic impulses may be controlled by cognitive capacity available to inhibit these impulses. Refusal self-efficacy is considered such a cognitive resource. Following this assumption, CBM interventions can yield benefits, especially for those with a relatively weak cognitive control over their impulses to drink [12,30-32]. In line with the predictions from dual process models and prior CBM trials [12], it is expected that for patients with a low baseline self-efficacy, the additional effect of the Alcohol Avoidance Training will be stronger. Similarly, time-varying self-efficacy is expected to partially mediate the CBM effect as repeated experiences of successful coping due to stronger avoidance responses will enhance refusal self-efficacy. Patients’ adherence and perceptions of treatment acceptability, and credibility will also be examined.

## Methods

### Trial Design

This study is a double-blind randomized placebo-controlled trial in a real-world setting. Patients receive TAU, consisting of outpatient personalized care

**Figure 1:**
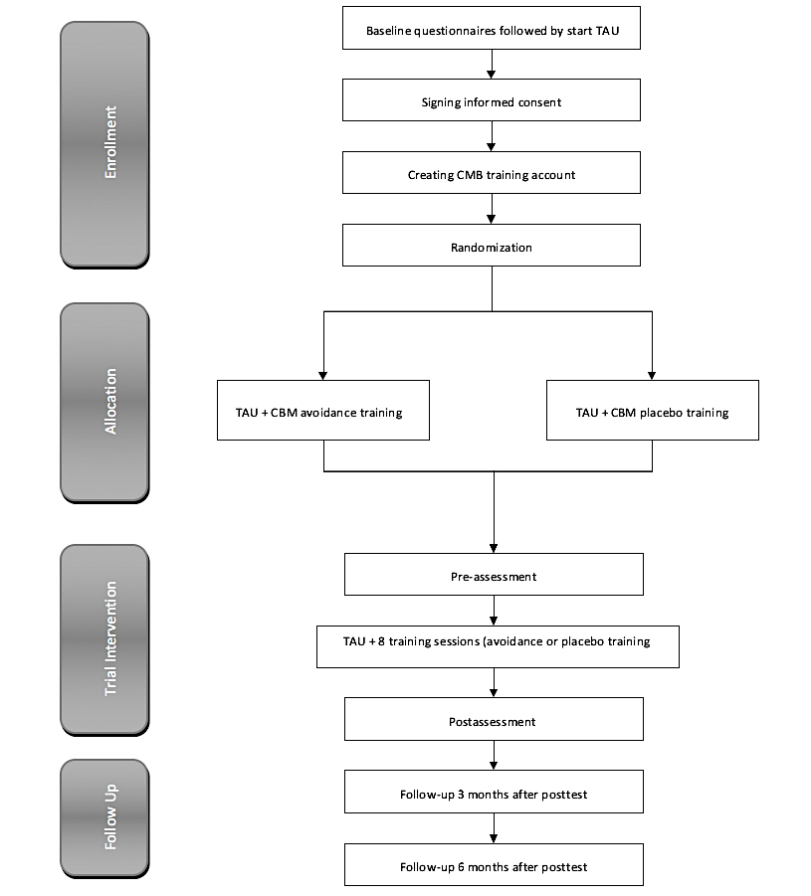
Participants flow chart. TAU: treatment as usual; CBM: Cognitive Bias Modification.

All patients with a primary alcohol problem enrolled for TAU are invited by their therapist to participate in the Alcohol Avoidance Training. After giving informed consent, patients are randomly assigned to one of the two training conditions: CBM Alcohol Avoidance Training or CBM placebo training. Patients begin the training simultaneously with the start of the behavioral change part of their treatment. Patients are recommended to follow a 15-minute CBM session twice a week for a period of five weeks. The CBM training includes eight sessions, preceded and followed by an assessment session, the preassessment and postassessment, respectively. Patients will be rewarded with a € 20 voucher if they complete all ten sessions. Three and six months after the TAU there will be follow-up assessments. Figure 1 shows the participant flow chart of the study.

The study has been approved by the Ethics Committee of Amsterdam Academic Medical Centre in January 2015 (reference number 2014_154#C20141463) and has been registered at the Netherlands Trial Register (NTR5087).

### Participants and Procedure

The study population consists of patients aged 18 years or older with a primary alcohol problem, who are currently following TAU at Tactus Addiction Treatment Institute in the Netherlands. One general inclusion criterion is accessibility and ability to use the Internet, since patients will need to access CBM-training online. Two exclusion criteria apply for the TAU: (1) serious psychiatric illness with a risk to decompensate while decreasing alcohol consumption; and (2) the possibility of severe physical illness as a consequence of decreased alcohol consumption. There are no additional criteria for participation in this study.

Patients for the training are recruited by therapists at Tactus Addiction Treatment Institute. After the regular intake procedure, including baseline questionnaires, the TAU starts. Before the patient reaches the goal-setting assignment, the therapist will inform the patient about the CBM training and provide the patient with further information about the study. If the patient wants to participate, an informed consent form will be provided to patients by the therapist. After signing the form, the patient receives login credentials for the CBM training from the researcher.

After finishing registration, the patient is randomly assigned to the Alcohol Avoidance Training or to the placebo training, and receives an email with a link to the CBM training website. After logging in, the patient receives instructions about the training. At the start of each session, the patient is asked to complete the additional questionnaires for the purpose of this study, consisting of self-reported weekly alcohol consumption and desire for drinking. Each of the eight training session takes about 10-15 min. The first training session is preceded by an (online) preassessment and the final training session is followed by an (online) postassessment. Three and six months after the postassessment, each patient will receive online follow-up questionnaires. In case of nonresponse, the patient will be reminded by email or phone to complete the questionnaire.

### Interventions

#### Treatment as Usual

TAU in this outpatient treatment setting is based on principles of CBT [33] and motivational interviewing [34]. The specific form of the TAU is tailored to the individual needs of the patient, in terms of treatment modality (Web-based or face-to-face) and intensity (brief 5-week version or intensive 3-month version). Study participants, therefore, receive an individualized version of treatment, as is common in regular real-world treatment settings. However, the basic ingredients for all versions are identical: daily registration, the analysis of the functions of the patients’ drinking behavior, behavioral change components, and motivational interviewing. Table 1 provides an overview of the main treatment ingredients. Sessions in face-to-face and Web-based treatment are identical. The only difference is that the contact with the therapist is synchronous in face-to-face treatment, and asynchronous via the Internet in the Web-based treatment [35]. The intensive version of the treatment takes approximately three months with (online or face-to-face) sessions once or twice a week and daily self-reporting of alcohol intake during the whole program. This treatment consists of two parts: a first part focusing on the analysis of the patients’ drinking habits, and a second part starting with the goal setting assignment, followed by sessions geared towards helping the patient reach the set goals and desired behavioral change. The brief 5-week version of the treatment focuses solely on behavioral change (part 2) and is intended for patients who already gained insight into their drinking habits when starting with treatment.

As we are interested in the effectiveness of online CBM Alcohol Avoidance Training as an adjunct to TAU, we do not differentiate between these four treatment “subgroups.” Due to the randomization, the experimental and control group are expected to be balanced concerning treatment modality and intensity.

Therapists have either a bachelor’s degree in social work or a master’s degree in psychology, and received a 2-day training on the treatment protocol of the TAU. Therapists can obtain expert advice from a multidisciplinary team consisting of treatment staff, an addiction physician specialized in addiction, a psychologist, and a supervisor. This multidisciplinary team also provides quality assurance through monitoring of client files and discussing treatment fidelity with counselors during weekly review of clients.

#### CBM Training

The intervention used in this study is the Alcohol Avoidance Training [21,23], based on the Approach-Avoidance Task (AAT) [36]. In this training, pictures of alcoholic beverages or soft drinks are presented, which are tilted 3 degrees to the left or right. Patients are instructed to respond to the tilted format of the picture, and not to the picture itself. This so-called irrelevant-feature version of the training (ie, responding to the format of the picture and not the subject of the picture itself) is used [21,23] because it is more indirect [37], and therefore blinds condition allocation (training vs placebo). Another advantage is that it is possible to change from measurement to training without changing the content of the picture set [38]. Striking the selected “u” key causes an avoidance movement of pictures in one format (eg, tilted left), while striking the selected “n” key causes an approach movement of pictures in the other format. The approach movement increases the size of the picture, and the avoidance movement decreases the size. This zooming effect generates a sensation of approach or avoidance, respectively. The combination of the format of the picture and the response (left=avoid and right=approach, vs left=avoid and right=pull) is counterbalanced across patients.

**Table 1:** Overview of the main treatment ingredients in intensive and brief treatments.

Session^a^	Content	Intensive (face-to-face/Web)	Brief (face-to-face/Web)
1	Baseline assessment	✓	✓
2	Advantages and disadvantages	✓	
3	Daily drinking diary	✓	
4	Description of drinking moments	✓	
5	Analysing drinking situations	✓	
6	Goal setting	✓	✓
7	Helpful thoughts	✓	✓
8	Helpful behavior	✓	✓
9	Decision moments	✓	✓
10	Action plan	✓	✓

^a^Part 1 is comprised of sessions 1-5 and part 2 is comprised of sessions 6-10.

**Figure 2:**
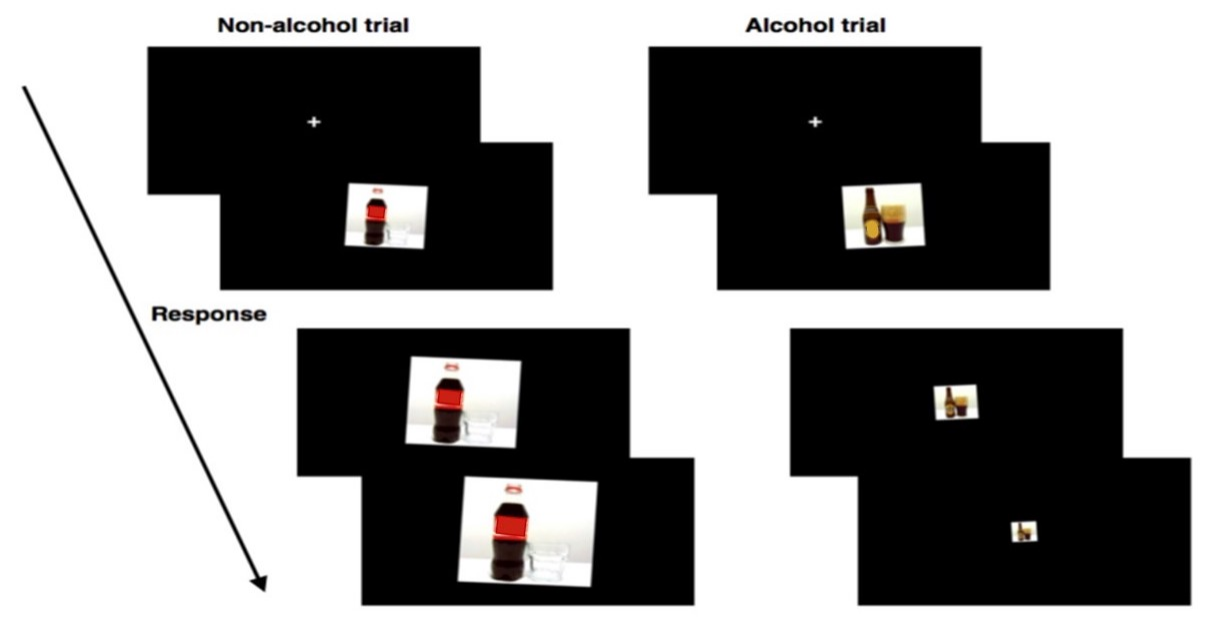
An example of Approach-Avoidance Training.

The complete training program consists of eight sessions. Each session starts with a practice block of 12 trials with gray squared pictures followed by 160 trials divided into 4 blocks. The use of blocks was adopted to make the task less monotonous and to provide a short break. Two sets (A and B) of 40 stimuli each are used of which 20 are for alcoholic beverages and 20 for soft drinks [39]. Patients randomly received either set A or set B for assessment and the other set for training to be able to test generalization to untrained stimuli. In the training condition, all 40 stimuli are repeated 4 times (alcoholic beverages in avoid format, soft drinks in approach format) to train patients to avoid alcohol by exposing them only to alcohol/push and soft drink/pull trials. In the placebo condition, all 40 stimuli are presented 4 times: 2 formats (tilted to the left or right) x 2 repetitions. Alcoholic beverages and soft drink pictures are presented equally often in both formats. Figure 2 shows an example of the Alcohol Avoidance Training. Stimuli stay on the screen for a maximum of 3000 ms. In the case of no response, the trial is restarted after repeating the instructions. Each trial starts with a fixation cross to keep patients’ attention focused.

### Measures

An overview of all measurements instruments along with the randomized controlled trial measurement time-points are presented in Table 2.

Demographic characteristics like gender, age, educational level, employment and clinical case history details (duration of alcohol dependence, previous detoxifications and treatments, duration of current abstinence and medication intake) will be collected during the baseline assessment of the TAU.

#### Alcohol Consumption

Weekly alcohol consumption will be assessed using the Alcohol Timeline Follow Back (TLFB) method [40]. For every day of the previous week, patients provide retrospective estimates on the number of standard units alcohol they consumed.

#### Alcohol Dependence

The type and severity of alcohol dependence will be assessed by using the Diagnostic and Statistical Manual of Mental Disorders IV criteria, by means of the Substance Abuse Module (SAM) of the Composite International Diagnostic Interview (CIDI) [41].

#### Craving

The 5-item Obsessive Compulsive Drinking Scale (OCDS) [42] assesses obsessionality and compulsivity related to craving and drinking behavior, and is derived from the original 14-item OCDS scale [43].

#### Health Status

Health status is evaluated using the Maudsley Addiction Profile, Health Symptom Scale (MAP-HSS). The MAP-HSS is a 10-item questionnaire that was adapted from the health scale of the Opiate Treatment Index [44]. Because the MAP-HSS measures only general physical complaints, eight additional alcohol-specific physical complaints were added: hyperventilation, sweating, diarrhoea, heart palpitations, headache, memory problems, sexual problems and epileptic seizures.

#### Depression, Anxiety and Stress

The 21-item Depression Anxiety Stress Scale (DASS-21)[45] is a self-report questionnaire designed to measure depression, anxiety and stress at baseline.

#### Drinking Motives

The baseline drinking motives will be measured using the modified Drinking Motives Questionnaire Revised (mDMQ-R) [46]. The mDMQ-R is a 28-item self-report inventory that assesses the frequency of drinking for each of the original four motives in Cooper’s model [47] with coping motives subdivided into coping-anxiety and coping-depression factors.

#### Self-Efficacy

Using eight items of the Drinking Refusal Self-efficacy Questionnaire (DRSEQ), participants will be asked whether they feel sure they can refuse alcohol on the three subdimensions of self-efficacy: social pressure, emotional relief and opportunism [48].

**Table 2:** Measurement instruments: purpose, measures and time points.

Purpose and measure^a^		Baseline TAU^b^	Preassessment		Training		Postasssessment		Posttest TAU		Follow-up	
**Cognitive bias assessment**												
	AAT			✓		✓		✓				
**Baseline measures**												
	Demographics	✓										
	MAP-HSS	✓								✓		✓
	DASS	✓								✓		✓
	OCDS	✓								✓		✓
	DMQ-R			✓								
	CIDI	✓										
	Drinking refusal self-efficacy	✓										
**Primary outcome measure**												
	Weekly alcohol consumption	✓		✓		✓		✓		✓		✓
**Secondary outcome measures**					✓							
	CEQ^c^					✓						
	CSQ							✓				

^a^AAT: Approach-Avoidance Task; MAP-HSS: Maudsley Addiction Profile; DASS: Depression Anxiety Stress Scale; OCDS: Obsessive Compulsive Drinking Scale Depression; DMQ-R: Drinking Motives Questionnaire (Revised); CIDI: Composite International Diagnostic Interview; CEQ: Credibilty Expectancy Questionnaire; CSQ: Client Satisfaction Questionnaire.

^b^TAU: treatment as usual.

^c^CEQ will take place in session 2.

#### Credibility of the Intervention

In the second training session intervention, credibility will be assessed using the Credibility and Expectancy Questionnaire (CEQ) [49]. It contains six items and differentiates between a patient’s thoughts and his or her feelings regarding the CBM training.

#### Client Satisfaction

The patient satisfaction regarding the CBM training will be assessed using the Client Satisfaction Questionnaire (CSQ) [50]. It contains 8 items and answers are given on a 4-point scale.

#### Approach-Avoidance Tendencies

Approach-avoidance tendencies are assessed with the AAT pre- and posttraining [23]. The tests consist of 172 trials; 12 practice trials (gray squared pictures) and 160 assessment trials, the latter subdivided into four blocks of trials. Participants randomly receive either set A or set B during assessment. Each set consists of 40 pictures (20 depicting alcoholic beverages and 20 soft drinks) and those are presented 4 times: 2 formats (tilted to the left or right) x 2 repetitions. Alcoholic beverages and soft drink pictures are presented equally often in both formats.

### Primary and Secondary Outcome Measures

The primary outcome measure for this study will be *the proportion of patients reporting alcohol consumption below low-risk drinking limits* (<22 standard units/week for men and <15 for women) [29], since achieving safe drinking is the primary aim of alcohol addiction care. This will be assessed using the TLFB method [40]. As it was not feasible to verify whether all participants met this threshold at the start of treatment and with the knowledge that a previous trial with a similar target group showed that enrolled participants only very rarely report below-threshold consumption levels at baseline [35]-, we allowed all patients to enroll in the study. If necessary, corrections will be made in the analyses.

Secondary outcome measures include changes in approach bias measured by an AAT in the preassessment and postassessment. An AAT bias index is calculated as the difference between the median reaction time scores for pushing pictures of one category (alcoholic beverages or soft drinks) and the median reaction time score for pulling pictures of that category. Median scores are used to minimize the influence of outliers. Positive scores indicate approach tendencies whereas negative scores indicate avoidance tendencies. Furthermore, it is investigated whether the added effect on treatment outcome is mediated by the amount of change in approach bias and who benefits most from training by identifying patient characteristics that moderate the outcome of the training. Other secondary outcome measures are general health condition, depression, anxiety and stress, intervention credibility and patient satisfaction.

### Randomization

Patients will be automatically assigned to one of the two conditions (Alcohol Avoidance Training or placebo training, as described in the Intervention section) with an equal likelihood, using the method of minimization [51] in order to balance for type of TAU (online vs face-to-face). The randomization will be computer-generated without any involvement by the investigators. Patients will be randomly allocated to the condition to which the fewest participants of that type of treatment have so far been assigned.

### Blinding

The trial has a double-blind design because neither patients nor therapists know to which condition patients are assigned. To ensure anonymity, patients receive an email with a user ID to create their personal research account. If necessary, patients can contact the researcher for help. Patients complete the training at their own computer. To keep patients blind to their intervention condition, patients respond to an irrelevant feature (orientation of the picture) instead of the content of the picture (alcoholic beverage vs soft drink beverage) [23]. During the postassessments, patients’ awareness of the experimental condition is assessed by means of a manipulation check.

### Sample Size Calculation

An a priori statistical power analysis (G-power) was conducted to determine the necessary number of participants. The primary outcome measure is a reduction in alcohol consumption. To obtain an estimate of the effect size to be expected, studies describing previous Alcohol Avoidance Training interventions were inspected [21,23]. In both these studies, a relative increase of 20% was observed in effectiveness of TAU + CBM as compared to TAU. The proportion of patients reaching long-term abstinence increased by 13% from 41%-54% [23]. Although in these studies a different dependent measure was used (prolonged abstinence from alcohol), we assume a similar increase in success rate of +13 percentage points as effect size. It has been shown that the Web-based treatment resulted in a 68% success rate (ie, reaching the low-risk drinking criterion) [52]. When extrapolating that to this study, a +13 percentage points additional effect of the Alcohol Avoidance Training is predicted to result in 81% achieving the safe drinking criterion (beta>.80; alpha (one-tailed) <.05). Based on these parameters, 152 patients are needed within a condition to show the hypothesized effects using a Fisher’s exact test for proportions in two independent samples.

### Statistical Analyses

Analyses will be conducted in agreement with intention-to-treat principle. Missing data points will be handled using multiple imputation [53]. One-way ANOVAs and χ²-tests will be performed to see if there are any significant differences at baseline between the two CBM training conditions (Avoid Alcohol vs placebo) for any of the demographic variables or outcome measures. Nonsignificant differences will indicate successful randomization. To determine the primary outcome, a logistic regression analysis will be used to test the differences in the proportion of patients reaching the low-risk drinking criterion between the CBM Alcohol Avoidance Training and CBM placebo condition, both at posttest and at follow-up. If necessary, baseline confounders will be controlled for, and significant interaction effects will be further investigated using post hoc *t*-tests (independent samples *t*-test and paired samples *t*-test). Effect sizes on clinically relevant outcomes at postintervention and follow-up will be calculated by Cohen’s d using the means and pooled standard deviations of the measurements of the conditions. Effect sizes of .56-1.2 are considered large, .33-.55 are considered moderate, and less than .33 are considered small [54]. Repeated measures ANOVAs will be conducted to test for differences between the CBM Alcohol Avoidance Training and the CBM placebo condition on the secondary outcomes general health condition, depression, anxiety and stress. Mediation analyses will be conducted by applying the analytic procedure according to Preacher and Hayes [55] and Hayes [56] to examine whether a change in approach bias is mediating the Alcohol Avoidance Training effects on our primary outcome measure. Logistic regression analyses will be used to assess whether patient characteristics moderate the effect of the Alcohol Avoidance Training on our primary outcome measure. The procedure proposed by Baron and Kenny [57] will be adopted. Descriptive statistics will be used to investigate to what extent patients adhered to the Alcohol Avoidance Training (in terms of timing, frequency, and duration of sessions) and to what extent patients found the Alcohol Avoidance Training both acceptable and credible (testing adherence, acceptability, credibility).

### Ethics, Consent, Permissions, and Funding

Written informed consent to participate in the study will be obtained from all participants.

The study has been approved by the Ethics Committee of Amsterdam Academic Medical Centre in January 2015 (reference number 2014_154#C20141463) and has been registered at the Netherlands Trial Register (NTR5087).

The study was funded by Saxion University of Applied Sciences, Enschede.

## Results

The trial was funded in 2014 and is currently in the active participant recruitment phase (started on May 2015). Enrolment will be completed in 2019. First results are expected to be submitted for publication in 2020.

## Discussion

This study protocol design describes a double-blind randomized controlled trial to assess the added value of an online Alcohol Avoidance Training as adjunct to TAU for outpatient alcohol patients. Previous studies involving alcohol dependent patients in inpatient addiction treatment have shown promising results for Alcohol Avoidance Training in addition to TAU [21,23]. The present study wants to test whether the positive added effects found in a more controlled, clinical setting, with training on local desktop computers, are replicated when the training is administered in a less controlled, ambulatory setting with online delivery.

The online delivery of CBM training enables patients to conduct the sessions at a preferred location, which may entail some threats to treatment fidelity. The preferred environment might bring distractions like ambient sounds or the interaction with other people present, which could influence the concentration level and responsiveness to the training [57]. Therefore, patients will be instructed to avoid distractions and that concentration, accuracy and speed during the sessions is required. Another aspect to take into consideration is that online interventions typically have a high dropout rate [58]. Therefore, treatment adherence is encouraged by: (1) emails from the research assistant to the therapists to enable them to monitor the progress of their patients when, for example, the patient has not finished a training session for some time; (2) emails or oral messages from the therapist to the patient to reinforce motivation; (3) emails to invite, remind and praise patients regularly. These messages are generated automatically by the training program whenever a patient can start a new training session, or when patients do not start with a new training session after the designated time; (4) a gift voucher of €20 from an online shop (Bol.com) when upon completion of all 10 study sessions (8 training sessions, and pre- and postassessment).

The strength of this study is the combination of online Alcohol Avoidance Training with TAU in an ambulatory setting. CBM training in an outpatient setting might be extra effective, because patients work on the training in their own relevant context with the presence of alcohol-related cues and challenges (eg, craving), that are not (or less) available in a clinical setting. So, patients can practice and apply their skills directly into the relevant setting. We are interested in investigating the impact of CBM training in the ambulatory setting. In addition, the ambulatory TAU is much less intensive than TAU in a clinical setting [23]. Therefore, the CBM training covers a proportionally greater part of the total treatment, and we will investigate whether this might have more impact. Additionally, the ambulatory and online delivery of the training will give us a first impression of the possibilities and concerns of the broader dissemination of CBM training.

## Abbreviations

AAT: Approach-Avoidance Task
CBT: cognitive behavioral therapy
CBM: Cognitive Bias Modification
DASS: Depression Anxiety Stress Scale
DMQ-R: Drinking Motives Questionnaire Revised
MAP-HSS: Maudsley Addiction Profile, Health Symptom Scale
mDMQ-R: modified Drinking Motives Questionnaire Revised
OCDS: Obsessive Compulsive Drinking Scale Depression
TAU: treatment as usual
TLFB: Time Line Follow Back
